# Mood variability during adolescent development and its relation to sleep and brain development

**DOI:** 10.1038/s41598-024-59227-9

**Published:** 2024-04-12

**Authors:** Yara J. Toenders, Renske van der Cruijsen, Jana Runze, Suzanne van de Groep, Lara Wierenga, Eveline A. Crone

**Affiliations:** 1 Developmental and Educational Psychology, Leiden University, Wassenaarseweg 52, 2333 AK Leiden, The Netherlands; 2 Leiden Institute for Brain and Cognition, Leiden University, Leiden, The Netherlands; 3 Department of Psychology, Education and Child Studies, Erasmus University Rotterdam, Rotterdam, The Netherlands; 4 Clinical Child and Family Studies, VU University Amsterdam, Amsterdam, The Netherlands

**Keywords:** Adolescence, Mood variability, Brain structure, Sleep, Actigraphy, Anxiety, Depression, Depression, Developmental biology, Circadian rhythms and sleep, Emotion

## Abstract

Mood swings, or mood variability, are associated with negative mental health outcomes. Since adolescence is a time when mood disorder onset peaks, mood variability during this time is of significant interest. Understanding biological factors that might be associated with mood variability, such as sleep and structural brain development, could elucidate the mechanisms underlying mood and anxiety disorders. Data from the longitudinal Leiden self-concept study (N = 191) over 5 yearly timepoints was used to study the association between sleep, brain structure, and mood variability in healthy adolescents aged 11–21 at baseline in this pre-registered study. Sleep was measured both objectively, using actigraphy, as well as subjectively, using a daily diary self-report. Negative mood variability was defined as day-to-day negative mood swings over a period of 5 days after an MRI scan. It was found that negative mood variability peaked in mid-adolescence in females while it linearly increased in males, and average negative mood showed a similar pattern. Sleep duration (subjective and objective) generally decreased throughout adolescence, with a larger decrease in males. Mood variability was not associated with sleep, but average negative mood was associated with lower self-reported energy. In addition, higher thickness in the dorsolateral prefrontal cortex (dlPFC) compared to same-age peers, suggesting a delayed thinning process, was associated with higher negative mood variability in early and mid-adolescence. Together, this study provides an insight into the development of mood variability and its association with brain structure.

## Introduction

Mood variability, or emotional instability, refers to fluctuations in mood over time^1^. Mood variability undergoes significant changes during adolescence^2^ and has been associated with negative mental health outcomes, such as an increased risk of developing anxiety and depression^3–7^. The goal of this study was to provide a comprehensive analysis of the developmental pattern of mood variability in adolescence and to examine two biological aspects that may be associated with these developmental patterns: sleep changes^8^ and structural brain development^9,10^.

Mood variability has previously been studied for both positive (e.g., happiness, vigor) and negative (e.g., sadness, anger, tension) emotions^2,11^. Mood variability is typically assessed using daily assessment of mood states, for example through self-report questionnaires, in which participants are asked to rate their mood across several days. This allows for the assessment of both general mood as well as daily fluctuations^12^. Mood variability is defined as the average difference in mood between consecutive days. Prior studies described several developmental patterns, although different methods were used. First, one prior study described that female adolescents (10–14 years at the first time point) showed an increase in mood variability over a 4 year period, when using standard deviation to calculate mood variability, suggesting a rise in mood variability in adolescence^13^. In contrast, a second longitudinal study in adolescents starting from the age of 13 years showed that mood variability for happiness, sadness and anger linearly decreased over a 5 year period in both boys and girls^2^. Furthermore, mood variability is an important outcome of emotion regulation, such that dysfunctional emotion regulation is thought to result in higher mood variability, and it was previously found that in mid-adolescence emotion regulation strategies were used less compared to early and late adolescence^14–16^. Emotion dysregulation is a characteristic of several psychiatric disorders, for example depression, that have their peak onset in adolescence^17,18^. Thus, the results on mood variability are mixed, but it could be that mood variability peaks in mid-adolescence, possibly associated with less efficient emotion regulation strategies, followed by a decrease into adulthood.

Prior studies have suggested an important link between mood and sleep^19,20^, with several longitudinal studies finding poor sleep is associated with concurrent low mood in adolescence^21,22^ and an increased risk for mental health difficulties later in life^22–26^. Changes in sleep patterns during adolescence may be related to shifts in melatonin release^8,27^, and the effects of social schedule, screentime, extracurricular activities and academic demands^28,29^. In addition, the build-up of homeostatic sleep pressure is more slowly in older adolescents^30^. These factors contribute to a decrease in total sleep duration, might cause sleep deprivation in adolescents, and has been assessed using both subjective and objective measures^19,31,32^. There is a discrepancy between objective and subjective sleep measures, and sleep measures remain to be optimized as self-reported sleep is often overestimated, and, while actigraphy is thought to be consistent it may underestimate sleep due to sleep-related motor activity, especially in boys due to their greater movements^32^. Therefore, it is of importance that the association between both measures of sleep and mood variability are assessed. Prior research showed that typically developing adolescents whose sleep was restricted to 6.5 h per night reported worse emotion regulation compared to adolescents who slept almost 9 h per night, but the relation with mood variability is still unclear^33^. Most prior studies examining concurrent mood and sleep were cross-sectional and included adolescents of a narrow age-range, which have impeded the ability to examine developmental changes. Longitudinal studies are therefore needed to study the association between the development of ‘natural’ sleep deprivation, which adolescents often experience, and mood variability^8^.

In addition to sleep related changes, structural brain development may be related to changes in mood and maturation of emotion regulation strategies^34^. During typical adolescent development, cortical thickness of the prefrontal cortex and other cortical regions consistently reduces^10,35,36^, and both delays and accelerations of this developmental process have been associated with symptoms of depression and anxiety^9,36–38^. Thicker cortical regions have been found in females, however, especially in early childhood^35^. Brain regions that are of particular interest for the relation to mood variability because of their involvement in emotion regulation include the prefrontal cortex (PFC), more specifically the ventrolateral (vlPFC) and dorsolateral (dlPFC) regions, as well as the anterior cingulate cortex (ACC), ventral striatum (VS), amygdala, and orbitofrontal cortex (OFC)^39–42^. Neuroscience models previously suggested that these regions do not develop simultaneously, as subcortical brain regions such as the amygdala and ventral striatum are thought to develop in a faster fashion than cortical brain regions such as the dlPFC and vlPFC^43,44^. It could be argued that this imbalance might lead to dysfunctional emotion regulation, therefore the association with mood variability is of interest.

Taken together, there is some evidence that mood variability is developing throughout adolescence^2,13^ and heightened levels of mood variability possibly have a negative effect on mental health^6,7^, but this question has not yet been examined using longitudinal measures across the whole range of adolescence. In addition, the association with two potential mechanisms that are also developing during adolescence, sleep, and neural development^19,38^, throughout the development of mood variability remains unknown*.*

Because of the simultaneous changes and the association with mental health difficulties, sleep and brain structure were examined to study the biological aspects associated with the development of mood variability. Studying biological aspects might provide further insight into the developmental mechanisms of mood variability.

Elucidating biological mechanisms of psychological risk-factors of mental health could contribute to our understanding of the development of mental disorders. Therefore, the present study had three aims: (1) to study the typical development of mood variability throughout adolescence in a longitudinal sample, (2) to study the relation between sleep, brain structure and mood variability throughout adolescent development, (3) to examine if mood variability during adolescence can predict anxiety and depressive symptoms. Because of the sex differences in mood and the development of depressive symptoms, the development of mood variability was studied per sex^2,45^.

We examined these questions in a pre-registered (https://osf.io/xmbg4/) comprehensive longitudinal study including three waves of neural development and daily variability in mood and sleep in adolescents between 11 and 24 years of age. We hypothesized that (a) mood variability develops in an inverted U-shape, (b) sleep decreases throughout development, (c) lower sleep length is associated with higher mood variability, (d) brain development of regions involved in emotion regulation is associated with mood variability, and (e) higher mood variability precedes symptoms of anxiety and depression. Additionally, an interaction analysis was preregistered that was not analysed due to the complex nature of this interaction (see Supplementary Material for details).

## Results

**Figure 1:**
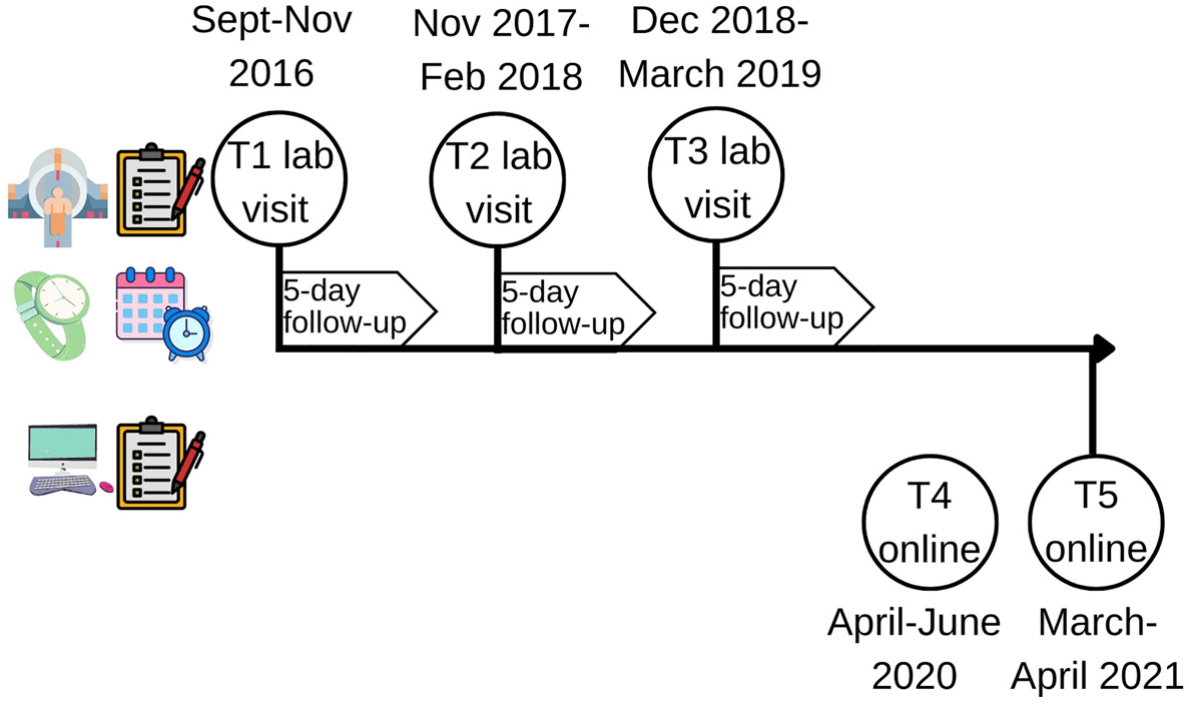
Timeline of the Leiden Self-Concept Study. At the first three time points, participants visited the lab to undergo an MRI and fill out questionnaires. In the 5 days after the lab visit, participants reported their daily mood and sleep in a diary, and sleep was measured using a wristband that detected motion. Participants were given the option to participate in two follow-up visits that only entailed filling out questionnaire measures at home.

Data from 191 participants over 5 time points were included, leading to a total of 661 observations (Fig. 1, Table 1, Supplementary Table S1).

**Table 1:** Overview participants over 5 time points.

	Time point 1 (N = 161)	Time point 2 (N = 168)	Time point 3 (N = 175)	Time point 4 (N = 116)	Time point 5 (N = 134)
Sex N (%)					
Male	71 (44.1%)	78 (46.4%)	81 (46.3%)	54 (46.6%)	63 (47.0%)
Female	83 (51.6%)	81 (48.2%)	88 (50.3%)	62 (53.4%)	71 (53.0%)
Age	15.40 (2.94)	16.01 (3.24)	17.59 (3.49)	18.92 (3.39)	20.27 (3.42)
Education N (%)					
Primary school	29 (18.0%)	23 (14.3%)	7 (4.0%)	4 (3.4%)	1 (0.7%)
High school	84 (52.8%)	87 (51.8%)	80 (45.7%)	47 (40.2%)	57 (42.2%)
Higher education	38 (24.2%)	46 (28.6%)	83 (47.4%)	65 (55.6%)	77 (57.0%)
Country of birth N (%)					
Netherlands	147 (91.3%)	152 (90.5%)	161 (92.0%)	107 (91.5%)	109 (80.7%)
Turkey	0 (0%)	0 (0%)	1 (0.6%)		0 (0%)
Netherlands Antilles	1 (0.6%)	1 (0.6%)	1 (0.6%)	1 (0.9%)	1 (0.7%)
Other	6 (3.7%)	6 (3.6%)	7 (4.0%)	2 (1.7%)	4 (3.0%)
Mood variability (negative)	8.41 (5.72)	8.93 (6.29)	9.39 (6.02)		
Average mood (negative)	10.82 (10.67)	12.19 (11.34)	13.54 (10.73)		
Mood variability	11.71 (6.26)	12.22 (7.16)	12.45 (6.54)		
Average mood	3.09 (12.25)	4.08 (12.65)	6.04 (12.71)		
Subjective sleep duration	9.26 (1.08)	9.16 (0.94)	9.02 (0.97)		
Energy level	2.89 (0.54)	2.83 (0.53)	2.70 (0.58)		
Objective sleep duration	7.73 (0.86)	7.58 (1.22)	7.35 (1.03)		
Sleep efficiency (%)	93.02 (5.63)	91.82 (8.13)	92.11 (7.61)		
Total anxiety symptoms	19.54 (11.75)	15.62 (9.99)	23.06 (14.39)	26.64 (13.77)	29.61 (16.54)
Total depressive symptoms	5.72 (3.74)	5.65 (3.95)	7.10 (4.53)	7.49 (4.65)	9.28 (5.79)

Mean (SD) are being displayed.

### Development of mood

The development of mood based on all five subscales of the profiles of mood states (POMS) has been reported in the Supplementary Material. The development of mood was further examined using only the four negative subscales of the POMS (tension, depression, anger, and fatigue), as it was found that the positive subscale (vigor) was not associated with age-related changes.

**Figure 2:**
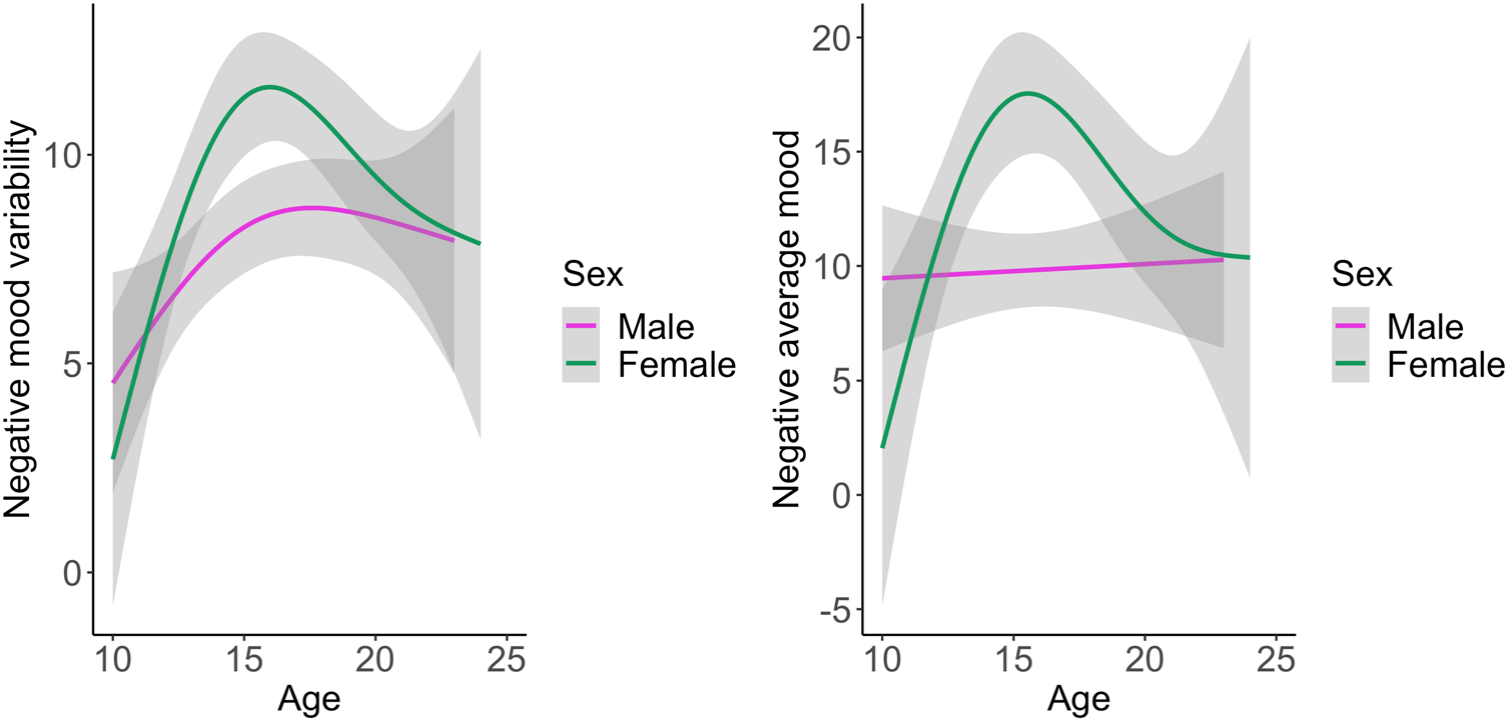
Best-fit model of negative average mood and negative mood variability development by sex. Left: negative mood variability, showing fluctuations in mood across 5 days. Right: average negative mood based in four POMS subscales (depression, anger, tension, and fatigue).

First, we tested for sex differences in average negative mood and negative mood variability using a linear mixed model. Negative mood variability is defined as the day-to-day change in negative mood^2^. This analysis showed that females (mean mood variability: 9.75 (SD: 6.44), mean average mood: 13.79 (SD: 11.60)) had higher negative mood variability and negative average mood than males (mean mood variability: 7.86 (SD: 5.18), mean average mood: 9.78 (SD: 9.37)) (F = 6.37, *p* = 0.012; F = 6.24, *p* = 0.014), therefore in all subsequent analyses the age by sex interaction was examined. Next, the development of negative mood variability was studied in an age by sex interaction model. The best-fit model on negative mood variability and negative average mood can be observed in Fig. 2 (*p* < 0.001, *k* = 4; *p* = 0.002). Negative mood variability showed a peak during mid-adolescence for females, showing a rapid increase and a modest decrease across adolescence (p = 0.02, *k* = 4; Fig. 2 and Supplementary Fig. S6). For males, negative mood variability increased throughout adolescence (p = 0.03, *k* = 4). Individual trajectories of mood variability are displayed in Supplemental Fig. S8. Negative average mood increased during early adolescence, also showed a peak during mid-adolescence and a decline during late adolescence for females but did not show an association with age in males (p < 0.001, *k* = 4; p = 0.66, *k* = 4).

### Development of sleep

**Figure 3:**
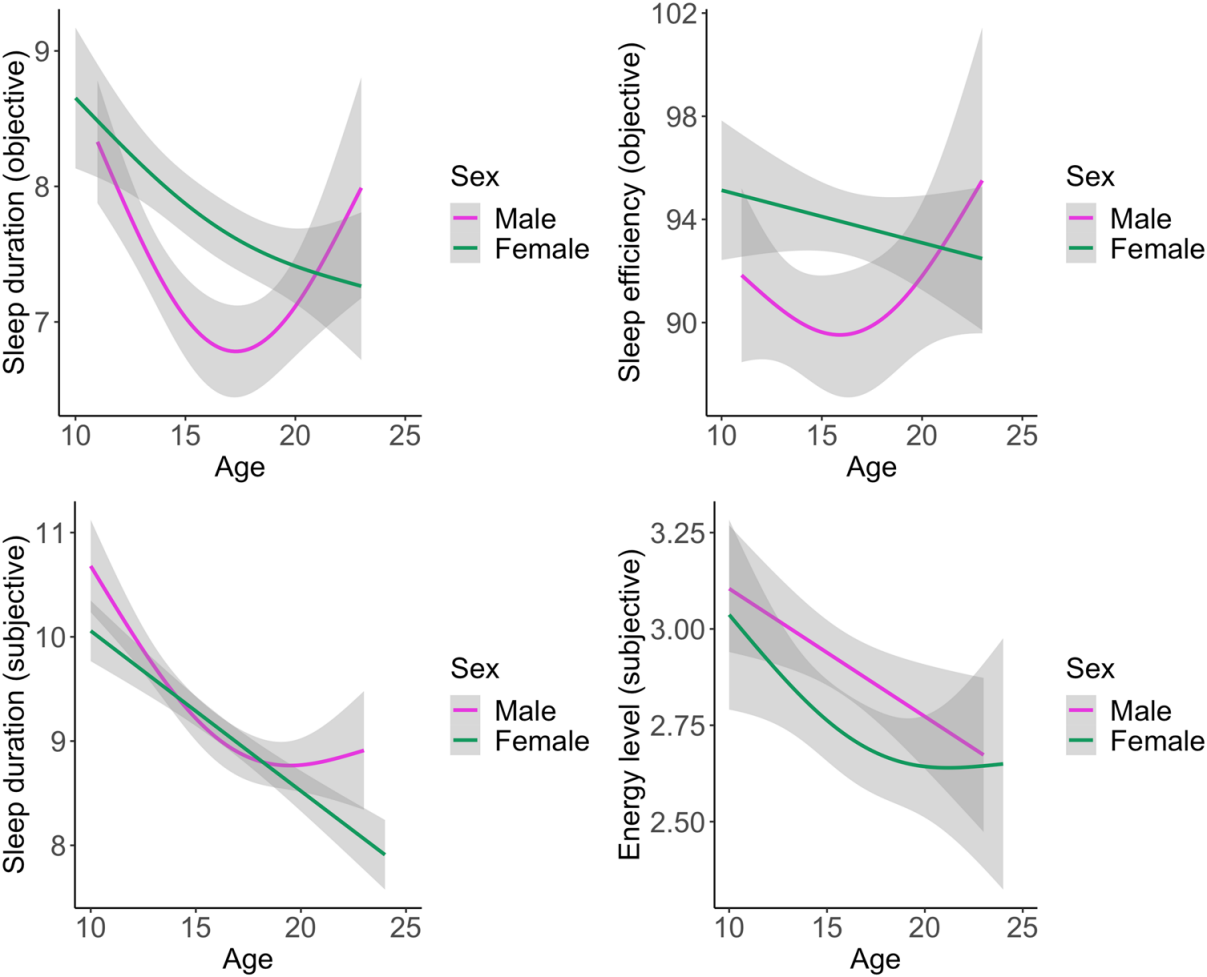
Best-fit model of sleep development by sex. Upper left: objective sleep duration, upper right: objective sleep efficiency, lower left: subjective sleep duration, lower right: energy level.

Next, the development of sleep: objective sleep duration, objective sleep efficiency (percentage asleep while in bed), subjective sleep duration and subjective energy level (measure of sleep quality), was investigated. The best-fit model for the age-by-sex interaction effects are displayed in Table 2, Fig. 3, and Supplementary Fig. S8.

**Table 2:** Best-fit model of sleep development. The best-fit GAMM models for the age-by-sex interaction to model the four measures of sleep are shown in the first three models (pFDR, k, and adjusted r2. Column 4–6 show the results of the GAMM models of the association between negative average mood and the sleep measures and column 7–9 show the results of the GAMM models of the association between negative mood variability and the sleep measures.

Sleep measure	Age-by-sex			Association with negative average mood			Association with negative mood variability		
	p_FDR_	k	Adjusted r^2^	p_FDR_	k	Adjusted r^2^	p_FDR_	k	Adjusted r^2^
Sleep duration (objective)	< 0.01*	3	0.23	0.50	3	0.72	0.91	3	0.57
Sleep efficiency	0.13	3	0.15	0.17	3	0.72	0.94	3	0.54
Sleep duration (subjective)	< 0.01*	3	0.47	0.68	3	0.54	0.94	3	0.65
Energy level	< 0.01*	3	0.33	< 0.01*	3	0.49	0.94	3	0.60

* p < .05.

There was a significant age-by-sex interaction for objective and subjective sleep duration, and subjective energy level but not for sleep efficiency. Objective and subjective sleep duration decreased for both males and females, but in males there was a subsequent flattening, and even increase, in late adolescence. Objective sleep efficiency did not show an age-by-sex interaction. However, subjective energy level decreased throughout adolescence, for both males and females.

### Association between sleep and mood throughout adolescence

**Figure 4:**
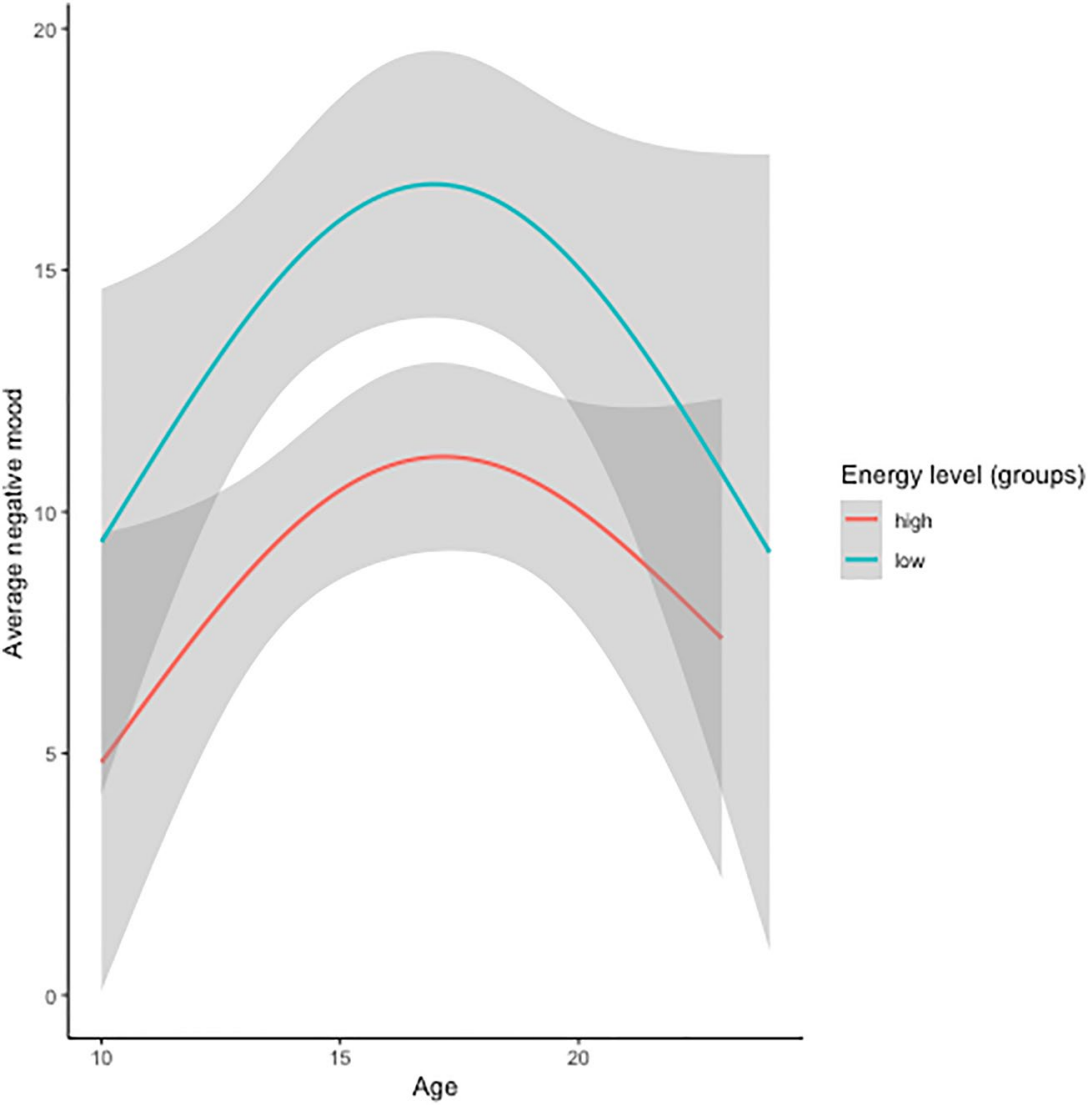
Mood variability development in groups with high or low levels of energy (subjective measure of sleep). Participants are merely divided into groups for visualisation purposes.

To study the association between sleep measures and mood, sleep measures were scaled to age. Sleep duration and sleep efficiency were not associated with negative mood variability or average mood. The best-fit models showed that age-corrected energy level and negative mood variability (p_FDR_ < 0.001, *k* = 3) showed an association, however, this association did not survive correction for average negative mood (Table 2). A second association was also observed between age-corrected energy level and average negative mood (p_FDR_ = 0.009, *k* = 3). People with lower level of energy compared to their age-matched peers showed higher negative mood throughout development (Fig. 4).

Subsequent control analyses confirmed that no effect of holidays was found, and the results did not change when the analyses were repeated separately for weekends and weekdays.

### Association between brain structure and mood throughout adolescence

**Figure 5:**
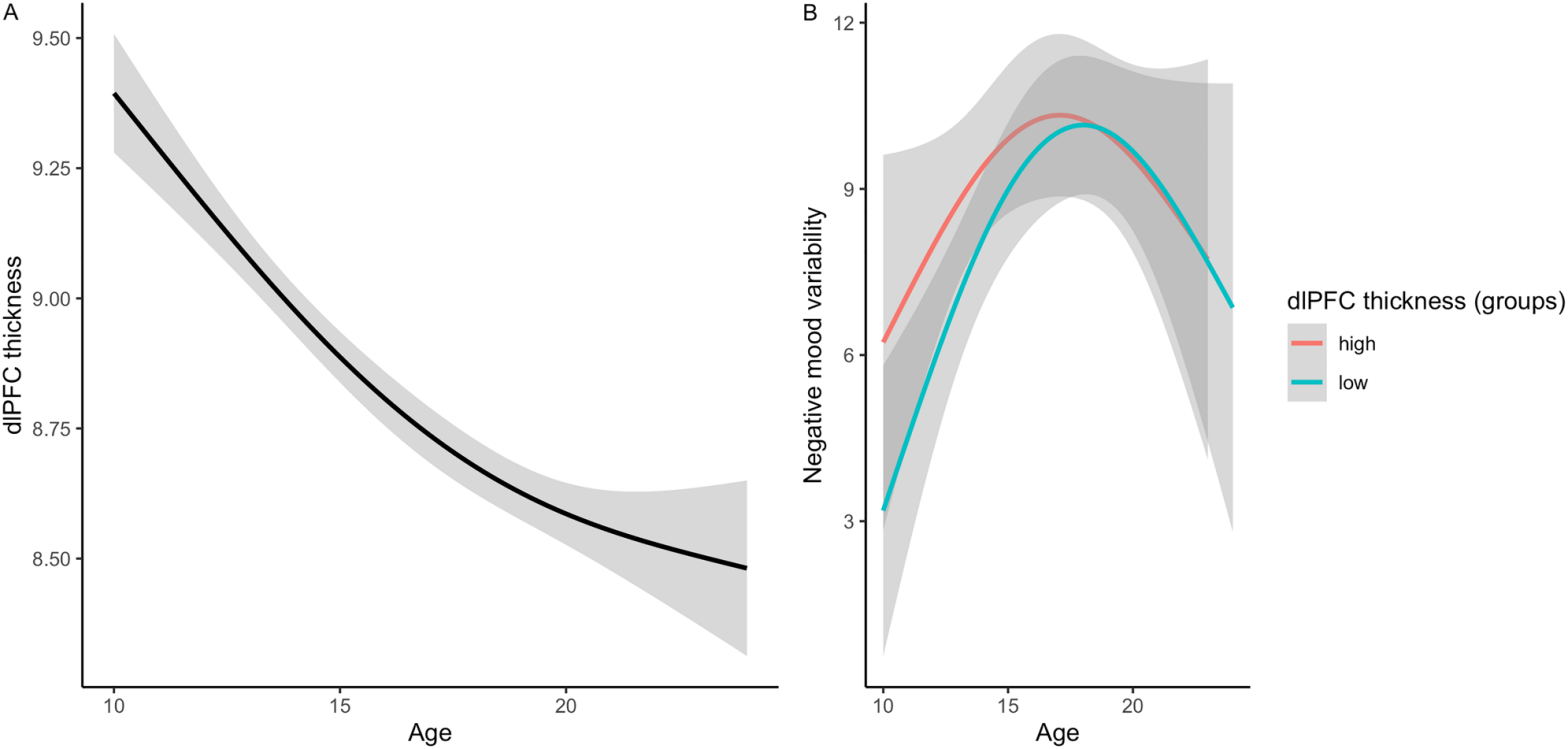
Development of dlPFC thickness (**A**) and mood variability development in groups with high or low dlPFC (**B**). Participants are merely divided into groups for visualisation purposes.

Next, the association between brain structure scaled to age and mood was examined. Consistent with prior studies^46,47^, cortical thickness for all regions decreased during adolescent development (for example: dlPFC in Fig. 5A).

Negative mood variability showed an association with dlPFC thickness (p_FDR_ = 0.04, *k* = 3, adjusted r^2^ = 0.60). Those participants with higher dlPFC thickness compared to same-age peers showed higher levels of negative mood variability in early and mid-adolescence (Fig. 5B). Negative average mood was not associated with brain structure. None of the other brain regions showed associations with negative mood variability or average mood.

### Association between mood variability and future anxiety and depressive symptomatology

The final aim was to investigate the association between mood variability and mental health, specifically whether mood variability preceded anxiety and depressive symptoms. It was found that mean mood variability at wave 1–3 preceded higher anxiety and depressive symptoms at wave 4 and 5 (depression: B = 0.32, p < 0.001; anxiety: B = 0.99, p < 0.001, Supplemental Fig. S10).

Finally, we tested whether this effect remained after correcting for average anxiety and depressive symptoms over wave 1–3, but it did not survive this correction (depression: B = 0.10, p = 0.16; anxiety: B = 0.26, p = 0.17). Correcting for age and sex did not change the results.

## Discussion

The present study used a longitudinal design to examine the development of day-to-day mood variability during adolescent development. As predicted, negative mood variability peaked in mid-adolescence, but only for females, whereas males showed linear increases in negative mood variability with age and lower levels of mood variability overall. Objective and subjective sleep duration dropped in mid-adolescence in line with prior research^31^ and males but not females showed a subsequent flattening and increase in sleep duration. Sleep duration was not associated with mood variability, but subjective energy levels were negatively associated with average mood (see also^19^). Lastly, consistent with prior research dorsolateral prefrontal cortex thickness declined with age^37^, and negative mood variability showed an association with the development of thickness in the dorsolateral prefrontal cortex.

Our first aim was to provide a longitudinal assessment of mood variability in adolescence. Due to the developmental patterns of mood observed (see Supplementary Material), we specifically focused on negative mood. The timing of the peak seen in mood variability was slightly later compared to previous research where a decrease was seen after age 13 years^2^. Additionally, this peak overlapped with the peak in average negative mood seen in females, showing that females not only showed larger mood swings but also an on average more negative mood in mid-adolescence. The current study adds to the literature as it completely covered adolescent development, and therefore the peak in mood variability can be observed in females. The development of mood variability was especially of interest because of its association with depression and anxiety, and onset of these psychiatric disorders peaks during adolescence^48^. This peak onset overlaps with the peak in mood variability. Additionally, prior research shows that biological aspects associated with mood and mood variability, sleep, and brain structure are also associated with mental health difficulties^9,22,23^. This might suggest that similar mechanisms, such as emotion dysregulation, might underlie the increase in mood variability and onset of psychopathology. In the current study the association between mood swings and future symptoms of depression and anxiety were specifically explained by current mood. It is important to note that these future mood disorder symptoms were measured during the COVID-19 pandemic^11^, which might have led to higher levels of depression and/or anxiety. While this might have increased the severity of the symptoms, it is not expected to have altered the direction of the results since symptoms before the pandemic were associated with symptoms during the pandemic.

The findings of a decrease in sleep duration (objective as well as subjective) fit well with prior research showing that there are sex differences in sleep development, and that overall, a decrease in sleep duration is observed^19,31^. Contrary to our predictions, neither objective nor subjective sleep duration were related to mood variability. Notably, developmental patterns associated with sex were inverted for males and females, with females showing a larger peak in mood variability and males showing a larger drop in objective sleep duration. These sex differences are currently not well understood, but some of these effects could potentially be related to social experiences and expectations. For example, males show higher levels of online gaming than females during adolescence^46^, possibly resulting in a shorter sleep duration in mid-adolescence. Alternatively, earlier research showed that adolescent females more often slept less than 6 or more than 10 h, which could have led to a higher average than in males^31^. Lastly, previous research showed that actigraphy less accurately measures sleep in males due to movement which might contribute to the large decrease in sleep observed^32^.

Despite the absence of a relation between mood variability and sleep and in line with previous research^21^, lower subjective energy levels, but not objective sleep measures, were associated with lower average mood. Even though energy levels decreased during adolescence, the relationship between average mood and energy levels did not change during development, suggesting that decreasing energy levels during adolescence co-occur with generally increasing negative mood. This potentially points to a causal relationship, therefore, it could be suggested that increasing sleep duration by for example adjusting school times could be beneficial for mood. However, these questions should be studies in future research using sleep intervention designs, as the current study, despite being longitudinal, is correlational and cannot draw causal conclusions.

One alternative explanation should be considered; subjective sleep has often been more strongly associated with depressed and anxious mood than objective sleep, which might be due to low mood being associated with the misperception of sleep, meaning that when people experience low mood, they often perceive their sleep as worse (Supplementary Table S4)^49,50^. This again highlights the need for intervention studies to explore the directionality of the association.

One of the mechanisms underlying the increased levels of mood variability in mid-adolescence might be emotion regulation^14^. Even though we did not measure emotion regulation strategies directly, we examined whether mood variability was associated with structural brain development of regions involved in emotion regulation^51^. A thicker dlPFC compared to same-age peers was associated with higher levels of negative mood variability in early and mid-adolescence, and a similar pattern was observed for mood variability and vlPFC thickness at trend level. Since adolescence is a time of cortical thinning, a thicker dlPFC could be interpreted as a delayed cortical maturation process compared to same-age peers. Thus, higher mood variability might be associated with a delay in cortical maturation. In late adolescence, after the age at which most rapid thinning of the dlPFC takes place^52^, the association with mood variability seems less pronounced. While this is the first study to show an association with mood variability, an earlier study, consistent with the current findings, showed that the development of dlPFC and vlPFC thickness was associated with the use of emotion regulation strategies, with greater cortical thinning associated with higher levels of cognitive reappraisal later in life^53^. Taken together, dlPFC thickness might be a biological marker that explains why some adolescents experience more mood swings than others.

Interestingly, no association between mood variability and limbic regions, the amygdala and ventral striatum, was found. This is however in line with work on the association between emotion regulation and subcortical development, also not reporting a direct association^54^. One possible explanation could be that subcortical regions do not show large intra-individual brain volume differences during adolescence and therefore no relation with developmental processes of mood variability^55,56^. Differences between surface area and thickness might explain why only an association between mood variability and thickness was found. For example, thickness is more susceptible to environmental influences whereas surface area is more strongly impacted by genetic factors^57^. In future research, examining the maturational coupling or functional activity or connectivity might provide more insight into the role of the subcortical regions in mood variability^58,59^.

A few limitations of the current study should be noted. First, the subjective sleep data was subject to recall bias, and the objective data to the adolescents wearing the actigraphy watch correctly. However, the strength of assessing both subjective as well as objective reports of sleep was that the relation of these two aspects of sleep to mood variability could be compared^49^. Besides sleep duration and quality, future studies could examine sleep variability, since it is associated with sleep quality, as well as brain connectivity^60^. It should be noted that we measured day-to-day variability of mood, whereas within-day variability might show a different pattern and different associations with biological mechanisms. We might not have picked up on the relation between mood variability and for example sleep because of the sampling rate of mood variability^61,62^. Future studies could directly measure emotion regulation and study the association with mood variability, as well as using a higher temporal resolution to measure mood variability and the effect of time when measuring mood variability by applying a method such as ecological momentary assessment (EMA)^63^. Since mood and energy show a diurnal pattern^64^, this could affect the relation with biological processes. Additionally, future (intervention) studies should explore the causal relation between the biological mechanisms and mood variability, as from this study it remains unknown whether they precede or follow mood swing development. Finally, the relation to pubertal hormone timing and development was not assessed. Since there were sex differences in the developmental trajectory of mood variability, and mood variability increased throughout puberty in females, hormone levels might play a role. Moreover, hormones have previously been related to brain development, and pubertal timing can affect the risk for developing psychopathology in adolescence^56,65^.

In conclusion, this study confirmed the hypothesis of increased levels of mood variability in adolescence with a peak in mid-adolescence, especially in females^2,13^, and that higher levels of mood variability are associated with future symptoms of anxiety and depression^6^. We found moderate evidence for a relation between average mood and subjective sleep energy levels, and small evidence for a relation between mood variability and structural brain development. Together, these findings suggest that mood variability, sleep duration changes, and neural development are co-occurring in adolescence. Yet, the study also highlighted sex differences which may be associated with different societal expectations and experiences. Future intervention studies should explore the causal relations between the biological mechanisms identified and mood swings. This study provides initial and important insight into the normative developmental patterns and biological aspects associated with risk factors for future depressive and anxiety symptoms.

## Methods and materials

### Participants

In this study, data from adolescents from the Leiden self-concept study was used^66,67^. The Leiden self-concept study is a longitudinal study with 5 time points. Participants were aged 11–21 years at the first time point and recruited from schools and online. They were followed for 3 consecutive years with lab visits, and 2 additional years with online questionnaires. The lab visits were followed by 5 days in which the participants filled out questionnaires on their mood and wore an actigraphy watch. Healthy adolescents (N = 191) took part in the study (Table 1, Supplementary Table S1). Participants were financially reimbursed. Exclusion criteria included the following: being left-handed, not having normal or corrected-to-normal vision, neurological or psychiatric diagnoses at T1, and usage of psychotropic medication. Participants and parents of participants younger than 18 years signed informed consent. The study (NL54510.058.16) was approved by the Medical Ethics Committee of the Leiden University Medical Centre (LUMC). All research was performed in accordance with relevant guidelines and regulations.

### Measures

#### Mood variability

The Profiles of Mood States (POMS) questionnaire was used to assess daily mood^68^. Participants answered these questions in an online questionnaire, and they received a notification at 6pm. Mood was measured at the end of the day, for participants to reflect on the day. In this questionnaire participants were asked to rate 32 adjectives on a 5-point Likert scale to which degree the adjective described their current mood. The POMS questionnaire consists of five subscales: anger, depression, fatigue, tension, and vigor. The participants filled out the questionnaire daily for a 5 day period after the lab visit. Mood variability was calculated per subscale as the absolute difference between successive days^2^. These scores per day were summed and then divided by the number of consecutive days the participants rated their mood. Total mood variability was defined as the sum of the mood variability on the subscales. Negative mood variability was calculated using the four negative subscales, excluding vigor. Only participants with ratings on 3 or more consecutive days were included. Higher scores indicate a higher mood variability. In addition, the total average mood was calculated by summing the averaged subscales and subtracting the average of the vigor subscale. Negative mood was calculated by summing the averaged negative subscales. Missing items were imputed using predictive mean matching from the ‘mice’ package in R^69^.

#### Sleep measures

Objective and subjective measures of sleep were included. Actigraphy watches were used to measure sleep duration and sleep efficiency objectively and using daily diaries subjective sleep duration and energy levels were assessed on the same days, for 5 days following the lab visit (Supplementary Methods).

#### Anxiety and depressive symptoms

During the lab visit, participants filled out the Revised Child Anxiety and Depression Scale (RCADS) to assess the participants’ feelings of anxiety and depression. Participants rated 47 questions on a Likert scale of 0–3. The answers were summed to create two subscales: total anxiety (sum of the anxiety subscales) and total depression^70^. Up to two missing items were allowed per subscale. Missing items were replaced by prorating the other items within the subscale.

#### MRI acquisition

MRI scans for the three waves were acquired on a Philips Ingenia 3.0 Tesla MR scanner. A standard whole-head coil was used. First, functional scans were obtained, followed by a high-resolution 3D T1-FFE scan (TR = 9.72 ms, TE = 4.6 ms, flip angle = 8°, 140 slices, voxel size = 0.875 × 0.875 × 0.875 mm, FOV = 224 × 178.5 × 168 mm). Participants watched a film while they were in the MRI.

#### MRI processing

The MRI data was processed in the longitudinal stream in FreeSurfer 6.0 (https://surfer.nmr.mgh.harvard.edu/)^71,72^. Parcellation of the cortex was based on the Desikan–Killiany atlas and Fischl atlas for subcortical regions^73,74^. Regions of interest were selected based on prior literature on emotion regulation. Cortical thickness and surface area of the following regions were included: dlPFC, vlPFC, ACC, and OFC, as well as volume of the following regions: ventral striatum and amygdala. The average of the left and right hemisphere was used.

The regions-of-interest were constructed by combining the following regions^9,55,75,76^:

dlPFC: superior frontal, rostral middle frontal cortex and caudle middle frontal, vlPFC: pars opercularis, pars triangularis and pars orbitalis, ACC: rostral ACC and caudal ACC, OFC: lateral orbitofrontal cortex and middle orbitofrontal cortex and ventral striatum: caudate, putamen and nucleus accumbens. This resulted in a total of six ROIs, which consisted of four cortical thickness, four cortical surface area, and two subcortical volume measures. The quality of the T1 images was assessed using Qoala-T^77^. Parts of these data were previously published^46,47^.

### Statistical analyses

Generalized additive mixed models (GAMMS) were used to study the development of mood variability and its association with sleep and brain structure. Generalized additive mixed models (GAMMs) are semi-parametric models that use penalized smoothing splines^78^ (Supplementary Methods).

First, an exploratory analysis was performed on the subscales (tension, anger, depression, fatigue, and vigor) to test for developmental patterns. Based on these findings, we created separate models for negative mood variability and average negative mood score, based on the first four subscales (see Supplementary Material). The main analyses (analyses 1–4 described below) were therefore done using these negative mood measures, as well as the general mood variability and average mood.

Data from the first three timepoints were used in the first four analyses, to study:

1. the development of mood variability (by testing the effect of age by sex on mood variability).
  $${Mood\, variability}_{ij}={\upbeta }_{0}+s1\left({Age}_{ij}\right){Sex}_{i}+{u}_{i}+{error}_{ij}$$
2. the development of sleep (by testing the effect of age by sex on sleep (separately for sleep duration, sleep efficiency, subjective sleep duration, energy level)).
  $${Sleep}_{ij}={\upbeta }_{0}+s1\left({Age}_{ij}\right){Sex}_{i}+{u}_{i}+{error}_{ij}$$
3. the association between mood variability and sleep (separately for each sleep measure, scaled by age) over time corrected for age and sex:
  $${Mood\, variability}_{ij}={\beta }_{0}+s1\left(z{Sleep}_{ij}\right)+s2\left({Age}_{ij}\right){Sex}_{i}+s3({zAverage\, Mood}_{ij})+{u}_{i}+{error}_{ij}$$
4. the association between mood variability and brain structure (scaled by age) over time corrected for age and sex.
  $${Mood\, variability}_{ij}={\beta }_{0}+s1\left({zROI}_{ij}\right)+s2\left({Age}_{ij}\right){Sex}_{i}+s3\left(z{AverageMood}_{ij}\right)+{u}_{i}+{error}_{ij}$$

The same analyses were repeated for average mood. In each analysis, i = subject, j = time point and ui = random effect per subject. The Benjamini–Hochberg false discovery rate (FDR) was used to correct for multiple comparisons with a significance level of 0.05. The analyses for mood variability and average mood were FDR corrected per analysis (1–4 above) to correct for multiple testing (so for example the association between mood variability and sleep was corrected for four tests). For analysis 4 it was corrected per type of brain measure (volume, surface area and thickness).

A thin plate spline was used to fit the data. Model fit, to choose the best *k*, was assessed using the BIC. *k* represents the basis dimensions, the maximum degrees of freedom for a smoothing spline. The ‘mgcv’ package in R was used for the analyses^79^. Participants were added as a random effect and restricted maximum likelihood (REML) was used for smoothness selection. The interaction effect of age and sex was included to test whether the development of mood variability differed between sexes. In analyses 3 and 4, sleep and brain structure were transformed into z-scores by scaling them to age. Participants with a large ROI or longer sleep duration at a certain age compared to their same-age peers had a higher z-score.

Several control analyses were conducted. The analyses for mood variability were corrected for average mood^2,12^. In addition, for analyses 2 and 3, the analyses were repeated with an additional covariate to correct for whether the participant was on holiday or at school/work. The analyses with objective sleep measurements were also repeated separately for weekend and weekdays.

To study the prediction of anxiety and depressive symptoms based on the development of mood variability, a linear regression was used. Average symptoms of anxiety and depression at follow-up (averaged over wave 4 and 5 because of the smaller sample size) was the dependent variable and the average level of mood variability (over wave 1, 2 and 3) was used as the independent variable. The analysis was corrected for the baseline level of anxiety and depressive symptoms by adding the average level of anxiety or depressive symptoms at the first three waves as a covariate. Additionally, age and sex at wave 1 were added as covariates in a sensitivity analysis.

### Supplementary Information

Supplementary Information.

## Data Availability

The data used is the study is available from the corresponding author upon request.
